# Hybrid genetic algorithm-based optimisation of the batch order picking in a dense mobile rack warehouse

**DOI:** 10.1371/journal.pone.0249543

**Published:** 2021-04-05

**Authors:** Jianglong Yang, Li Zhou, Huwei Liu

**Affiliations:** 1 School of Management Engineering, Capital University of Economics and Business, Beijing, China; 2 School of Information, Beijing Wuzi University, Beijing, China; Chongqing Jiaotong University, CHINA

## Abstract

The utilization of a storage space can be considerably improved by using dense mobile racks. However, it is necessary to perform an optimisation study on the order picking to reduce the time cost as much as possible. According to the channel location information that needs to be sorted, the multiple orders are divided into different batches by using hierarchical clustering. On this basis, a mathematical model for the virtual order clusters formed in the batches is established to optimize the order cluster picking and rack position movement, with the minimum picking time as the objective. For this model, a hybrid genetic algorithm is designed, and the characteristics of the different examples and solution algorithms are further analysed to provide a reference for the solution of the order picking optimisation problem in a dense mobile rack warehouse.

## Introduction

Warehousing activities, as a key link in the field of logistics, considerably influence the logistics industry. In the face of current market trends that require improved storage conditions, industrial changes due to the technological advances and pressures such as land rents and labour costs, dense warehousing systems have gradually become the focus of the industry. In fields such as medical logistics, cold supply chain logistics, and domains involving highly demanding storage conditions, the cost of constructing such a storage system is considerably high. In some core areas of commercial prosperity and densely populated cities, dense storage systems are often present to maximise the utilisation efficiency of the storage space. Due to the high cost of rent, it is necessary to consider the use of dense storage systems to improve the space utilisation and reduce the storage costs per unit of the storage space.

**Fig 1:**
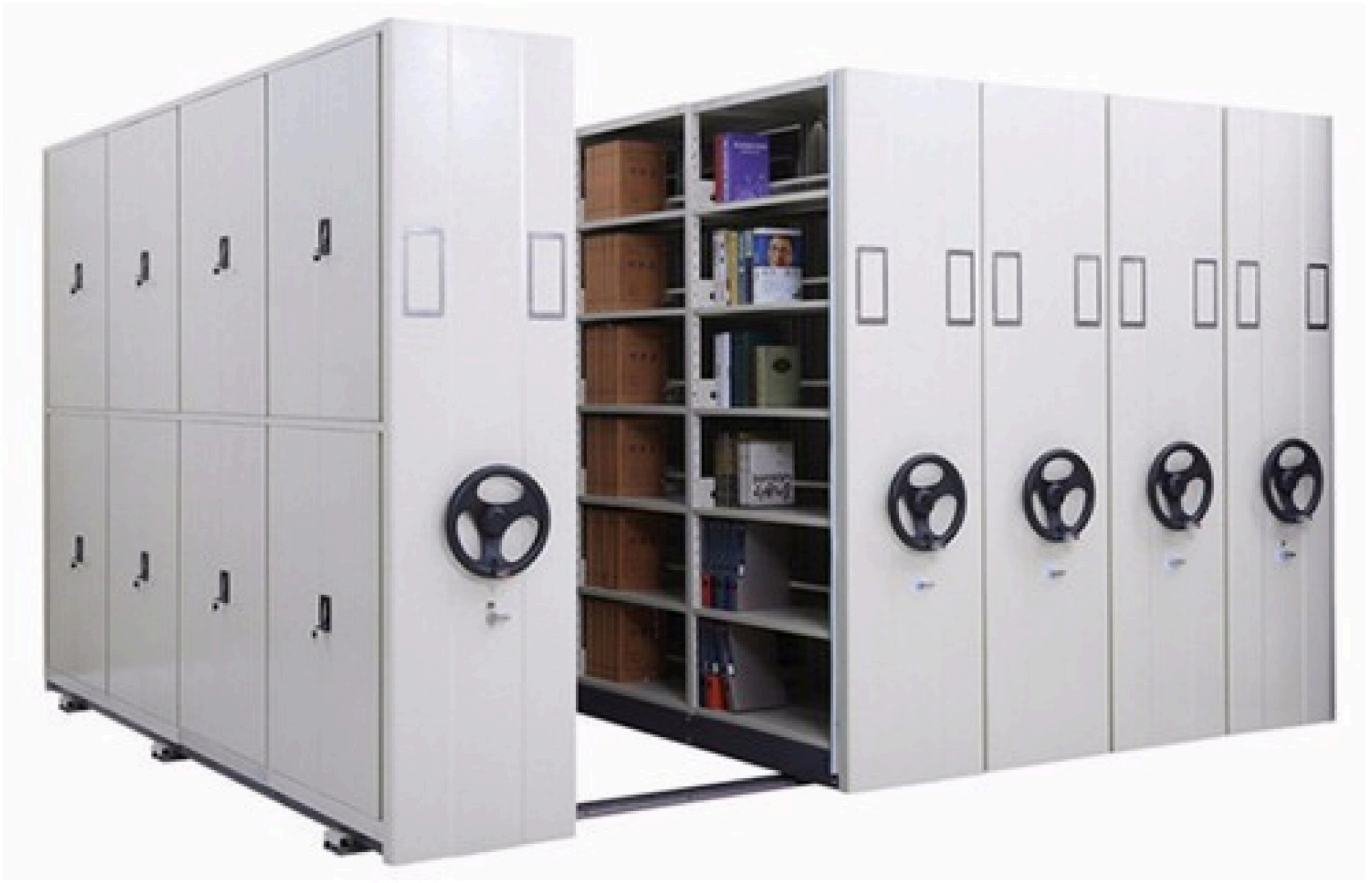
Schematic diagram of the dense mobile racks.

Among different types of dense storage systems, dense mobile racks are attracting increasing attention. Due to the simple structure, flexible disassembly and assembly, convenient operation, and associated significant improvement in space utilisation, such systems can be applied to expend the warehousing practices. As shown in Fig 1, a dense mobile rack has a compact overall structure and is installed on the rails laid on the ground; each row of racks can move laterally along the rails. In such a dense mobile rack storage system, one or more channels are reserved, and the position of the channel changes with the movement of the rack; consequently, the goods stored in different rack positions can be sorted.

The dense mobile rack storage system considerably improves the utilisation efficiency of the storage space; however, in the picking process, the racks must be moved and different channel positions must be opened, leading to a deterioration in the picking result. In particular, when picking multiple orders, if the order picking or rack movement is different, the efficiency of the picking activities may be further reduced, thereby affecting the overall storage operation. Therefore, it is necessary to optimise the efficiency of the picking activities and reduce the time consumption of the goods outbound operations by determining the appropriate order picking and rack movement. Based on this, this study:

1. In order to solve this problem, a multi-channel order picking model for dense mobile shelves is established, and the nature of the problem is further discussed;
2. The optimization algorithm of hybrid genetic algorithm and greedy algorithm is designed, and a new index of the termination condition of hybrid genetic algorithm is proposed to measure the optimization of the solution more clearly;
3. Through the simulation of examples, the influence of parameters on the nature of the problem is analyzed and different application situations are analyzed.

## Research status of dense mobile rack storage systems

The research on the order picking problem of an intensive storage system mainly focuses on three aspects: The first aspect is the analysis of the characteristics of the intensive storage and the design of a new dense storage system, the second aspect is the study of the order picking in the intensive storage system, and the third aspect is the development of a feasibility algorithm for the order picking problem in a dense storage environment.

There exist different types of dense warehousing systems in terms of the design and picking characteristics. Li L [1] proposed a special intensive warehousing system, which is characterised by moving the bins that store the goods on the racks and arranging the picking on the path of the bins. Kaukl CV et al. [2] designed a multi-dimensional automatic warehousing picking system, in which each cargo consists of multiple drives that fit into the rack rails and push the cargo to slide along the rack rails, thereby making the multi-dimensional automatic warehousing efficient and considerably improving the utilisation of the storage space. Yugang Yu, René BM de Koster et al. [3] performed research on a special compact system with a rotary conveyor for a deep movement and warehouse picking (S/R) system that could enable the horizontal and vertical movement of the unit loads. Nima Zaerpour, Yugang Yu et al. [4] studied a next-generation storage system, namely, the live-cube compact storage system and derived the estimated closed-form formula for the sorting time under two types of classification storage to evaluate the performance of any configuration and system boundary at the first zone. Subsequently, the size and regional boundaries of the live-cube compact storage system under two types of classified storage were further optimised to minimise the picking time and reduce the order response time [5]. Venkat R et al. [6] studied a new jigsaw-based approach for the dense storage and proposed a heuristic algorithm for the worst-case boundary for large-scale load picking vehicles.

Due to the characteristics of dense warehousing, the problem of order picking in dense warehousing systems has always been the focus of research. While improving the space utilisation rate, we must also design an optimisation plan for order picking to reduce the picking time as much as possible to optimise the picking efficiency. Bo Qinghe [7] analysed the movable and layered rotary rack storage systems and studied the picking operation activity as a special type of the travelling salesperson problem (TSP); the mathematical model of the path optimisation was solved using the ant colony algorithm. Reilly PJ et al. [8] studied the access mechanism of a unit cargo in a dense storage system; these researchers used the discrete Markov chain to quantify the position of a unit cargo and establish the access mechanism function under an increase in the uncertainty. Boysen N et al. [9] explored the order picking queuing problem in mobile rack warehouses and optimized the order queue to reduce the time of moving the heavy racks and enhance the picking efficiency. Masoud Mirzaeia et al. [10] constructed an optimal walking path for multiple loads and suggested multi-load, multi-empty slots as a future research direction.

In the research of the feasibility algorithm, the order picking problem for a dense storage system mainly involves the heuristic algorithm, although the genetic algorithm finds a relatively higher application. Liu Quanwei [11] used a multi group genetic algorithm(MGGA) to study the problem of the automatic storage location allocation and task scheduling for a single carrier and multi carrier. Meng Chunhui [12] studied the task scheduling problem of dense automated warehouses, analysed the running trajectory of the stacker, established the mathematical model with the shortest working time as the target, and solved the problem by using a genetic algorithm(GA). Nastasi G et al. [13] compared the optimisation results of the niched Pareto genetic algorithm(NPGA), non-dominated sorting genetic algorithm(NSGA) and strength Pareto genetic algorithm(SPGA) in the automated warehousing strategy, and the results indicated that the non-dominated sorting genetic algorithm is significantly better than the other two algorithms. In addition, the combination of NSGA and Clarke-Wright saving method is applied to minimize the total generalized cost of location-routing problem [14]. Ning Zhao et al. [15] designed a shuttle-based warehouse picking system (SBS/RS) and proposed a collision-free lift trajectory prediction acceleration/deceleration method combined with a collision-free method by using the GA to achieve the sequencing and assignment of the order picking. Imahori and Hase [16] studied the SKU allocation and the optimal sequence of order retrieval in A-type warehouse system to minimize the total retrieval time. They analyzed the computational complexity and developed a graph-based heuristic algorithm(GHA) to obtain the optimal sequence for order retrieval and SKU allocation.

A brief summary of the existing research results is shown in Table 1. At present, the research on the intensive warehousing mainly focuses on the abovementioned three aspects, and research on the intensive mobile racks is relatively rare. In particular, when one or more movable channels exist in a dense mobile rack storage system, the order picking situation becomes more complicated. For the multiple random phenomena involved in the order picking process, further modelling and analysis are needed. The greedy algorithm and genetic algorithm are commonly used to solve such problems; however, different algorithms can also be employed, such as the hybrid of genetic algorithm and simulated annealing algorithm [17]. The objective is to estimate the order picking time under the different picking rules to explore the nature of the problem and compare the characteristics of the different algorithms.

**Table 1:** Brief summary of existing research.

Types of dense storage system	System optimization scheme	Feasibility algorithm		
		Research issues	Algorithm	Objective function
Intensive system [1] Multi-dimensional automatic system [2] Compact storage system [3] Live-cube compact system [4, 5] Jigsaw-based dense system [6]	Path optimization [7, 10] Location access optimization [8] Order queuing optimization [9]	Location allocation & task scheduling [11]	MGGA	Min total operation time
		Task scheduling problem [12]	GA	Min operation time
		Multi objective optimization [13]	NPGA, NSGA and SPGA	Max total storage weight & space, and min damage
		Location-routing problem [14]	NSGA based on Clarke-Wright	Min total generalized cost
		Order sequencing and scheduling [15]	GA	Min collision & avoidance
		Operation planning and scheduling [16]	GHA	Min total retrieval time

## Order batch picking for a dense mobile rack

### Problem description and variable hypothesis

**Fig 2:**
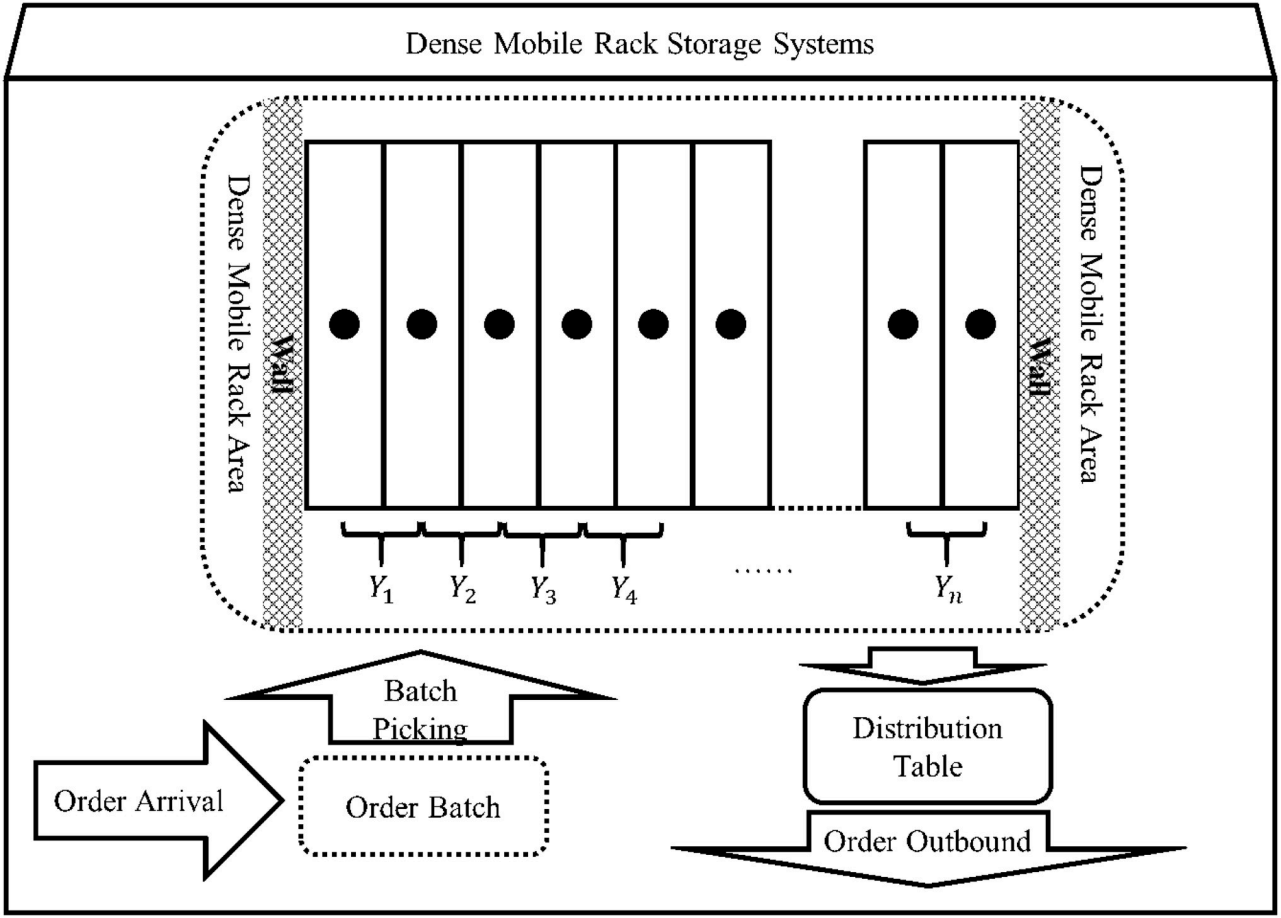
Dense mobile rack storage system.

Suppose there exists a set of dense mobile racks. As shown in Fig 2, the set of moving channel numbers that can be opened between the racks is {1, 2, …,n}, and the corresponding interval is *Y* = [1, *n*]. In this case, *Y*_*j*_ = *j*, where *j* = 1, 2, …, *n*.

In an actual dense mobile rack storage system, to pick the goods, the position of the rack must be moved and the target goods must be picked from the open channels. The moving speed of the rack is 4m per minute, and the speed of the picker or the picking vehicle is 115m per minute. It can be seen that the picker or the picking vehicle is faster than the rack movement. Therefore, the time taken for the rack movement is mainly considered in the model, and the moving time of the picker or picking vehicle is neglected.

In the dense mobile rack storage system, the channel position is moved by moving the rack. Assuming that the channel position moves from *j* to *j* + 1 or *j* − 1, it is necessary to move the adjacent row of racks by a unit distance, which has a time cost of c units. If the channel moves further, the time taken is the corresponding multiple of the moving unit distance.

### Assumption of the order picking

Assuming that the processing method of the order picking is batch processing according to the inspection cycle, the *Q* orders that arrive within a certain inspection period are collectively selected. In an actual warehousing operation, not all the orders are selected one by one. According to the channel that needs to be opened, the orders of the same or similar channels are merged into one batch for unified picking. Subsequently, through the distribution station, multiple orders are split into different batches, and finally, the goods are sorted according to the goods demand of each order. In the batch picking, a lesser number of batches corresponds to a faster picking process. However, according to the specific conditions of the picking and dispensing equipment of the storage system, it is impossible to unify all the goods into one batch picking. Therefore, the number of batches to be divided *v*(1 < *v* < *Q*) should be considered according to the actual situation.

If *O* represents the order set, *O* = {*O*_1_, *O*_2_, …, *O*_*Q*_}. By considering the information of the item to be picked up in each order and comparing the storage location information of the goods in the warehouse, the channel to be opened for each order can be further determined. The mobile channel that needs to be opened corresponding to the *d* − *th* order *O*_*d*_ can be represented as a corresponding picking information vector (*X*_*d*1_, *X*_*d*2_, …, *X*_*dn*_), where 1 ≤ *d* ≤ *Q*, 1 ≤ *j* ≤ *n*.

$$\begin{array}{c} X_{dj}=\{\begin{array}{cc} 1 & Open\;the\;jth\;channel\\ 0 & Do\;not\;open\;the\;jth\;channel \end{array}, \end{array}$$ (1)

In the inspection cycle, the moving channels that need to be opened for the *Q* orders can be represented by the following matrix ***X*** of *Q* × *n* orders, where each row of the matrix ***X*** represents the picking information of an order.

$$\begin{array}{c} X=[\begin{array}{cc} \begin{array}{cc} X_{11} & X_{12}\\ X_{21} & X_{22} \end{array} & \begin{array}{cc} \cdots & X_{1n}\\ \cdots & X_{2n} \end{array}\\ \begin{array}{cc} \vdots & \vdots \\ X_{Q1} & X_{Q2} \end{array} & \begin{array}{cc} \ddots & \vdots \\ \cdots & X_{Qn} \end{array} \end{array}], \end{array}$$ (2)

### Order batching based on the hierarchical clustering

It is assumed that *Q* orders are batched, and the order set of the *k* − *th* batch is *H*_*k*_, where *D*_*pq*_. The degree of difference 1 ≤ *k* ≤ *Q* − *v* refers to the minimum difference between the number of *H*_*k*_ channels to be opened and the number of channels to be opened for order *O*_*p*_ or *O*_*q*_ when *H*_*k*_ = {*O*_*p*_, *O*_*q*_}.

$$\begin{array}{c} D_{pq}=min\left\{\sum_{j=1}^{n}\frac{1}{2}\left[|X_{pj}-X_{qj}|-\left(X_{pj}-X_{qj}\right)\right],\sum_{j=1}^{n}\frac{1}{2}\left[|X_{qj}-X_{pj}|-\left(X_{qj}-X_{pj}\right)\right]\right\}, \end{array}$$ (3)

Thus, each order is treated as a cluster, and the degree of difference between any two clusters can be obtained. In addition, the difference matrix of *Q* orders can be obtained.

$$\begin{array}{c} D^{1}=[\begin{array}{cccc} D_{11} & D_{12} & \cdots & D_{1Q}\\ D_{21} & D_{22} & \cdots & D_{2Q}\\ \vdots & \vdots & \ddots & \vdots \\ D_{Q1} & D_{Q2} & \cdots & D_{QQ} \end{array}], \end{array}$$ (4)

where *D*_*dd*_ = 0, *d* = 1, 2, …, *Q*, and the matrix ***D*** is symmetric about the main diagonal element; i.e., *D*_*pq*_ = *D*_*qp*_, where *p* ≠ *q*.

According to the difference degree list, using the hierarchical clustering method, the *Q* orders can be divided into several batches via the following process:

**First clustering**.The smallest element of the matrix ***D***^1^ is searched except in the main diagonal lower triangle position; the row and column in which the smallest element is located is determined. Next, the orders corresponding to the row and column are merged into one cluster. The first cluster is defined as:

$$\begin{array}{c} D_{pq}=min\left(D^{1}\right), \end{array}$$ (5)

0here *p* ≠ *q*, and *p* ≥ *q*. Consequently, *H*_1_ = {*O*_*p*_, *O*_*q*_}.

Next, the picking information vector of a new virtual order cluster is determined as:

$$\begin{array}{c} B_{1}=\left(max\left\{X_{q1},X_{p1}\right\},max\left\{X_{q2},X_{p2}\right\},\cdots ,max\left\{X_{qn},X_{pn}\right\}\right), \end{array}$$ (6)

If *max*{*X*_*q*1_, *X*_*p*1_} = *b*_11_, *max*{*X*_*q*2_, *X*_*p*2_} = *b*_12_, …, *max*{*X*_*qn*_, *X*_*pn*_} = *b*_1*n*_, *B*_1_ = (*b*_11_, *b*_12_, …, *b*_1*n*_).

**Second clustering**. *B*_1_ is used as a virtual order after the *O*_*p*_ and *O*_*q*_ channel information is merged. The difference between *B*_1_ and the other clusters except *O*_*p*_ and *O*_*q*_ is determined, and a new difference matrix ***D***^2^ is formed. After searching for the smallest element of the triangle position of ***D***^2^, the orders corresponding to the row and column are merged into one cluster (*H*_3_), and the second cluster is completed.

**Fig 3:**
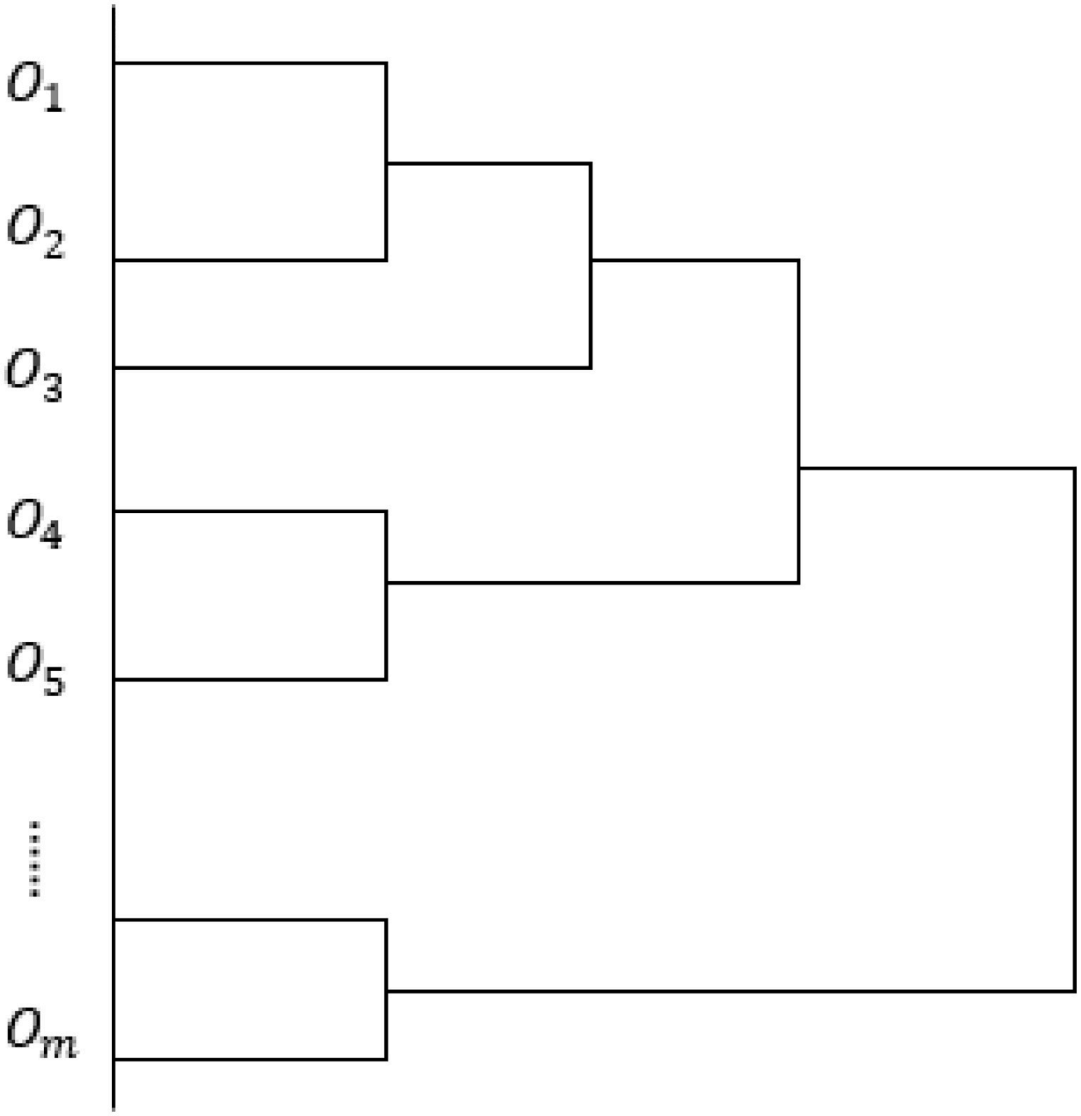
Schematic diagram of the order clustering process.

In a similar manner, all the pair of clusters are combined to obtain a new virtual order cluster until all the clusters are finally gathered into one large cluster. The schematic diagram of the clustering process is shown in Fig 3. This process involves (*Q* − 1) clustering processes, and the values of (*H*_1_, *H*_2_, …, *H*_*Q*−1_) can be obtained. Subsequently, the specific information of the order batch can be obtained, and the *Q* orders are divided into different batches from 1 to *Q*, under various clustering scenarios for the considered situation.

According to the specific facility conditions of the different storage systems and order picking requirements, the specific batch quantity can be determined. When the orders are divided into *v* batches for sorting, the picking scheme contains *v* clusters, which can be regarded as *v* virtual order clusters (or initial order picking information vectors). The picking information vectors of the *v* order clusters can be represented as a *v* × *n* matrix ***B***, and each matrix ***B*** can act as the sorting information for a virtual order cluster.

$$\begin{array}{c} B=[\begin{array}{cccc} b_{11} & b_{12} & \cdots & b_{1n}\\ b_{21} & b_{22} & \cdots & b_{2n}\\ \vdots & \vdots & \ddots & \vdots \\ b_{v1} & b_{v2} & \cdots & b_{vn} \end{array}], \end{array}$$ (7)

The channel position that needs to be opened for the virtual order cluster *B*_*i*_ can be expressed as a non-zero element in the set {*b*_*i*1_ ⋅ *Y*_1_, *b*_*i*2_ ⋅ *Y*_2_, …, *b*_*in*_ ⋅ *Y*_*n*_}, where *b*_*ij*_ ⋅ *Y*_*j*_ ≠ 0, 1 ≤ *i* ≤ *v*.

***Definition 1***: The “Picking active interval” is $\left[b_{i\cdot }^{min}Y_{\cdot },b_{i\cdot }^{max}Y_{\cdot }\right]$; the minimum non-zero element in the set {*b*_*i*1_ ⋅ *Y*_1_, *b*_*i*2_ ⋅ *Y*_2_, …, *b*_*in*_ ⋅ *Y*_*n*_} is the lower limit of the interval, which is recorded as $b_{i\cdot }^{min}Y_{\cdot }$, and the largest non-zero element is considered as the upper limit of the interval, which is denoted as $b_{i\cdot }^{max}Y_{\cdot }$. For example, the vector of the virtual order cluster picking information is (0, 1, 0, 0, 0, 1, 1, 0), and the corresponding picking channel number set is {0, 2, 0, 0, 0, 6, 7, 0} therefore, the picking active interval is [2, 7].

## Order batch picking model of a dense mobile rack

In an intensive mobile shelf storage system, the order batch picking model selects each order cluster sequentially for the order information of the *v* order clusters, based on the abovementioned order batching. According to the actual investigation of a storage system, in a warehouse picking system, the critical link affecting the efficiency of the picking activity is a part of the picking process. For the batch picking, after the picking activity is completed, the time taken to dismantle the goods is less when the order included in each order cluster is implemented. Therefore, this model does not consider the time spent on the distribution. In addition, in a dense mobile racking system, there may exist one or more channels. This paper considers a single channel as a special case of the multi-channel system for the modelling analysis. Although the model is designed for multi-channel situations, it is equally applicable in the case of a single channel. Combined with the above description of the dense mobile rack order batch picking situation, the variable assumptions and model are established as follows:

1. *x*_*i*_: the order cluster number of the *i* − *th* pick;
2. $R_{j}^{x_{i}}$: the channel number of the *j* − *th* movement selected in the order cluster $B_{x_{i}}$;
3. *E*: the total number of channels present in the mobile rack;
4. *e*^*j*^: the channel number of the *j* − *th* selection movement;
5. $T_{j}^{e^{j}}$: the position number of the *e*^*j*^ − *th* channel before the *j* − *th* movement; the initial position of the *E* moving channels is given as $\left\{T_{1}^{1},T_{1}^{2},\ldots ,T_{1}^{E}\right\}$.

The objective function is the minimum total time spent on the order picking, and the model is established as follows:

$$\begin{array}{c} \begin{array}{c} \begin{array}{cc} min & z=\sum_{i=1}^{m}\sum_{j=1}^{m}|T_{j}^{e^{j}}-R_{j}^{x_{i}}|\cdot c \end{array}\\ \left\{ \begin{array}{c} b_{x_{i}R_{j}^{x_{i}}}Y_{R_{j}^{x_{i}}}\neq 0;Y_{R_{j}^{x_{i}}}=R_{j}^{x_{i}}\\ T_{j+1}^{e^{j}}=R_{j}^{x_{i}};T_{j+1}^{f}=T_{j}^{f}\\ x_{i}\neq x_{\varepsilon },i\neq \varepsilon \\ R_{j}^{x_{i}}\neq R_{\theta }^{x_{i}}\neq 0,j\neq \theta \\ 1\leq x_{i}\leq v;1\leq R_{j}^{x_{i}}\leq n\\ i,\varepsilon =1,2,\cdots ,v;j,\theta =1,2,\cdots ,n\\ 1\leq E\leq n,1\leq e^{j}\leq E;1\leq f\leq E,f\neq e^{j}\\ b_{x_{i}R_{j}^{x_{i}}}=0or1;x_{i},R_{j}^{x_{i}}\geq 0\text{andareintegers} \end{array}\right. \end{array}, \end{array}$$ (8)

## Design of the hybrid genetic algorithm

In the algorithm design, the greedy rules are designed according to the nature of the problem model, and the preliminary solution is formed by the greedy algorithm. Since the greedy rules are determined, the calculation results with a higher optimisation degree can be obtained through fewer calculation steps. Compared with that of the genetic algorithm that can achieve the same optimisation level, the speed of the greedy algorithm is higher and the calculation process is more certain. However, due to the limitations of the greedy rules, the greedy algorithm has only a limited degree of optimisation for which the solutions can be obtained, and the genetic algorithm must be used to obtain a solution with a higher optimisation level. Therefore, the corresponding hybrid genetic algorithm is designed, and the solution result of the greedy algorithm is used as the initial group, which can make the genetic algorithm reach a higher level of optimisation at the beginning of the iterative operation.

### Design of the greedy algorithm

#### Greedy rule

***Definition 2***: The “channel invalid moving time” corresponds to the channel first moving to the upper or lower limit of the picking active interval and later moving in the opposite direction to traverse all the picking positions. Considering the initial position of the channel, the time that can be saved by employing the different movement directions (lines) is recorded as $W_{i}^{x_{i}}$, which indicates the channel invalid moving time obtained for the order $O_{x_{i}}$ at the *i* − *th* calculation.

According to the least channel moving time in the selection process of the virtual order cluster, the order to be picked is determined. In addition, a multi-channel configuration can be converted into multiple single-channel cases for the purpose of this research.

**Fig 4:**
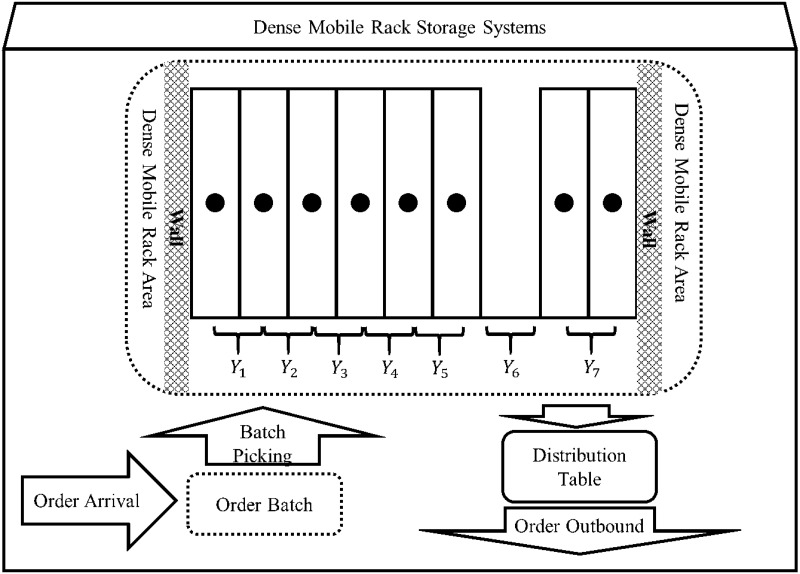
Channel movement analysis.

As shown in Fig 4, a group of mobile racks involves 7 channel positions (the racks next to the wall are fixed racks are cannot be moved) when only one channel is available at position 6, and the 0 − 1 vector of the order picking information is (1, 1, 0, 0, 0, 1, 1). To complete the order picking, several options are available:

1. The channel first moves from the 6*th* position to the 7*th* position on the right, and it later moves to the 1st position on the left, traversing the position of the non-zero element in the order picking vector; the time spent is (7 ⋅ *c*);
2. The channel moves to the 1*st* position on the left and later moves to the 7*th* position on the right, traversing the non-zero position of the order vector; the time spent is (11 ⋅ *c*);
3. The channel traverses the position of each non-zero element in the virtual order cluster picking information vector in a random order; however, this process requires more time.

In the picking process of the considered order, the most fundamental purpose is to traverse all the picking positions in the least time. If the initial position of the channel is 1 or 7, the complete picking process needs to only move directly to the right or left to traverse the complete set. The picking active interval can be used, and the total time is (6 ⋅ *c*).

***Definition 3***: The “best route of passage” corresponds to the following route: according to the principle of the shortest invalid moving time of the channel, the channel first moves to the upper or lower limit of the nearest picking active interval and later moves to the other end of the picking active interval to traverse all the positions to be picked. If the channel is already at the upper or lower limit of the picking active interval, it moves directly to the other end. The time taken for the best movement route of the channel of the *i* − *th* virtual order cluster is the time taken for the *i* − *th* virtual order cluster picking, which is recorded as *J*_*i*_.

#### Picking situation

**Single channel situation** In the case of a single channel, the virtual order cluster to be picked is determined for the *i* − *th* time, and the current single mobile channel position is defined as $\left\{T_{i}^{1}\right\}$. Here, 1 ≤ *i* ≤ *v*, and $T_{i}^{1}$ can be determined by the initial position of the moving channel and the optimal moving route of the channel selected by each virtual order cluster. According to the virtual order cluster picking information matrix ***B***, the channel invalid moving time of each virtual order cluster under the current moving channel position can be calculated.

Without a loss of generality, when the virtual order cluster to be selected is determined for the *i* − *th* time, the virtual order cluster that has not been selected is {*B*_1_, *B*_2_, …, *B*_*v* − *i*+1_}. The total number of virtual order clusters that have not been selected is (*v* − *i* + 1), and the invalid movement time of each virtual order cluster is as follows:

$$\begin{array}{c} \begin{array}{c} W_{1}^{i}=min\left\{|T_{i}^{1}-b_{1,\cdot }^{min}Y_{\cdot }|,|T_{i}^{1}-b_{1,\cdot }^{max}Y_{\cdot }|\right\}\\ W_{2}^{i}=min\left\{|T_{i}^{1}-b_{2,\cdot }^{min}Y_{\cdot }|,|T_{i}^{1}-b_{2,\cdot }^{max}Y_{\cdot }|\right\}\\ \vdots \\ W_{v-i+1}^{i}=min\left\{|T_{i}^{1}-b_{v-i+1,\cdot }^{min}Y_{\cdot }|,|T_{i}^{1}-b_{v-i+1,\cdot }^{max}Y_{\cdot }|\right\} \end{array}, \end{array}$$ (9)

According to the principle that the channel has the shortest moving time, the *i* − *th* selected virtual order cluster is

$$\begin{array}{c} B_{x_{i}}=\left\{B_{i}|\min\left\{W_{1}^{i},W_{1}^{i},\cdots ,W_{v-i+1}^{i}\right\}\right\}, \end{array}$$ (10)

Among these values, if multiple minimum values exist, a branching calculation must be performed.

The picking time of the virtual order cluster $B_{x_{i}}$ is $J_{x_{i}}=\left(b_{x_{i},\cdot }^{max}Y_{\cdot }-b_{x_{i},\cdot }^{min}Y_{\cdot }+W_{x_{i}}^{i}\right)\cdot c$.

Therefore, the total time for an m virtual order cluster picking is $z=\sum_{i=1}^{v}J_{x_{i}}$.

*Multi-channel situation*. According to the number of channels, the racks are sorted and selected. When *E* channels are present, the racks can be divided into *E* areas. According to the principle that the channel has the shortest moving time, the channels move in each area, thereby making a virtual order. Consequently, the clusters are sorted one by one.

In the case of *E* channels, the virtual order cluster to be picked is determined for the *i* − *th* time, and the current mobile channel positions are known, which are recorded as $\left\{T_{i}^{1},T_{i}^{2},\ldots ,T_{i}^{E}\right\}$. In this case, 1 ≤ *i* ≤ *v*, 1 ≤ *e* ≤ *E*, and $T_{i}^{e}$ can be determined according to the initial position of the moving channel and the optimal moving route of the channel selected by each virtual order cluster.

According to the current channel position, the interval *Y* = [1, *n*] corresponding to the rack position is divided into *E* sub-regions, which are represented by intervals and are recorded as $\left\{U_{i}^{1},U_{i}^{2},\ldots ,U_{i}^{E}\right\}$; subsequently,

$$\begin{array}{c} U_{i}^{1}=\left[1,\lfloor \frac{T_{i}^{1}+T_{i}^{2}}{2}\rfloor \right];U_{i}^{2}=\left[\lceil \frac{T_{i}^{1}+T_{i}^{2}}{2}\rceil ,\lfloor \frac{T_{i}^{2}+T_{i}^{3}}{2}\rfloor \right];\cdots ;U_{i}^{E}=\left[\lceil \frac{T_{i}^{E-1}+T_{i}^{E}}{2}\rceil ,n\right], \end{array}$$ (11)

In this expression, ⌊ ⌋ denotes rounding down, and ⌈ ⌉ denotes rounding up.

According to the virtual order cluster picking information matrix ***B***, the picking active interval in each sub-interval of the *i* − *th* virtual order cluster is recorded as $\left\{\left[b_{U_{i}^{1},\cdot }^{min}Y_{\cdot },b_{U_{i}^{1},\cdot }^{max}Y_{\cdot }\right],\left[b_{U_{i}^{2},\cdot }^{min}Y_{\cdot },b_{U_{i}^{2},\cdot }^{max}Y_{\cdot }\right],\ldots ,\left[b_{U_{i}^{E},\cdot }^{min}Y_{\cdot },b_{U_{i}^{E},\cdot }^{max}Y_{\cdot }\right]\right\}$.

When a picking active interval is an empty set $\left(b_{U_{i}^{e},\cdot }^{min}Y_{\cdot }=b_{U_{i}^{e},\cdot }^{max}Y_{\cdot }\right)$, to avoid influencing the sum calculation of the virtual order cluster channel moving time, let $b_{U_{i}^{e},\cdot }^{min}Y_{\cdot }=b_{U_{i}^{e},\cdot }^{max}Y_{\cdot }=T_{i}^{e}$.

Without a loss of generality, when the virtual order cluster to be selected is determined for the *i* − *th* time, the virtual order cluster that has not been selected is {*B*_1_, *B*_2_, …, *B*_*v*−*i*+1_}. The total number of virtual order clusters that have not been selected is (*v* − *i* + 1). Calculating the sum of the invalid channel movement times of each virtual order cluster in each divided position sub-interval under the current mobile channel position, the invalid movement time of each virtual order cluster is as follows:

$$\begin{array}{c} \begin{array}{c} W_{1}^{i}=\sum_{e=1}^{E}min\{|T_{i}^{e}-b_{U_{1}^{e},\cdot }^{min}Y_{\cdot }|,|T_{i}^{e}-b_{U_{1}^{e},\cdot }^{max}Y_{\cdot }|\}\\ W_{2}^{i}=\sum_{e=1}^{E}min\{|T_{i}^{e}-b_{U_{2}^{e},\cdot }^{min}Y_{\cdot }|,|T_{i}^{e}-b_{U_{2}^{e},\cdot }^{max}Y_{\cdot }|\}\\ \vdots \\ W_{v-i+1}^{i}=\sum_{e=1}^{E}min\{|T_{i}^{e}-b_{U_{v-i+1}^{e},\cdot }^{min}Y_{\cdot }|,|T_{i}^{e}-b_{U_{v-i+1}^{e},\cdot }^{max}Y_{\cdot }|\} \end{array}, \end{array}$$ (12)

According to the principle that the sum of the invalid movement times of the channels in each sub-interval of each virtual order cluster is the smallest, the *i* − *th* selected virtual order cluster is:

$$\begin{array}{c} B_{x_{i}}=\left\{B_{i}|min\left\{W_{1}^{i},W_{2}^{i},\cdots ,W_{v-i+1}^{i}\right\}\right\}, \end{array}$$ (13)

If multiple minimum values exist, a branching calculation must be performed. The picking time for order $B_{x_{i}}$ is $J_{x_{i}}=c\cdot \sum_{e=1}^{E}\left(b_{U_{x_{i}}^{e},\cdot }^{max}Y_{\cdot }-b_{U_{x_{i}}^{e},\cdot }^{min}Y_{\cdot }+W_{x_{i}}^{i}\right)$. Therefore, the total time for a v virtual order cluster picking is $z=\sum_{i=1}^{v}J_{x_{i}}$.

### Design of the genetic algorithm

Although the greedy algorithm can be used to determine the selection order and channel movement route of the optimal virtual order cluster in a short time, the optimisation degree of the solution is limited. Therefore, based on the greedy algorithm, the genetic algorithm is designed to enhance the degree of optimisation of the solution. In addition, this algorithm design is applicable to both single-channel and multi-channel situations.

#### Code

According to the nature of the problem, three random characteristics are considered in the process of order picking: First, the sequence of picking *v* orders can be randomly arranged; second, the sequence of picking the different channel positions in each order can be randomly arranged; third, the location of the channel before the order picking influences the order picking time, and the channel for sorting can be randomly selected amongst multiple channels. Therefore, the method of a “real matrix pair” is used to perform the individual coding; each individual is a “matrix pair” containing two real matrices, and it is denoted as *F*.

$$\begin{array}{c} F=\{A,B\}, \end{array}$$ (14)

where ***A*** is the moving channel numbering matrix in the greedy algorithm calculation result, and ***B*** is the order picking sequence matrix in the greedy algorithm calculation result; consequently, $A={\left[a_{ij}\right]}_{v\times n}$, and $B={\left[b_{ij}\right]}_{v\times n}$.

In addition, the separate order picking sequence set $O_{s}=\left\{O_{s_{1}},O_{s_{2}},\ldots ,O_{s_{v}}\right\}$ is used as auxiliary code for cross operation, where *s*_1_, *s*_2_, …, *s*_*v*_ ∈ {1, 2, …, *v*}.

#### Generate an initial group

According to the calculation result of the coding rule and the greedy algorithm, the values of the corresponding elements in the matrix ***A*** and ***B*** are determined. Next, the *g* results of the greedy algorithm are copied as the initial individual, which is recorded as {*F*_1_, *F*_2_,…,*F*_g_} = {{***A***_1_, ***B***_1_}, {***A***_1_, ***B***_1_},…,{***A***_g_, ***B***_g_}}.

In the iterative calculation process, any individual can contain the sequence of the virtual order clusters, the sequence of the channel positions selected by each virtual order cluster, and all the information regarding the movement of the channels for the picking.

The auxiliary coding information is recorded as $\{O_{s}^{1},O_{s}^{2},\ldots O_{s}^{g}\}=\left\{\left\{O_{s_{1}}^{1},O_{s_{2}}^{1},\ldots ,O_{s_{v}}^{1}\right\},\left\{O_{s_{1}}^{2},O_{s_{2}}^{2},\ldots ,O_{s_{v}}^{2}\right\},\ldots ,\left\{O_{s_{1}}^{g},O_{s_{2}}^{g},\ldots ,O_{s_{v}}^{g}\right\}\right\}$.

#### Determine the fitness function

The design of fitness function is closely related to the setting of selection operator. In this study, roulette is used for individual selection, that is, individuals with high fitness are selected first. It is necessary to change the objective function from small optimization direction to large optimization direction, and ensure that the result is positive, then it can be used as fitness function. Therefore, according to the objective function of the order picking model, this study takes the difference between the maximum value of the group objective function and the objective function value of each individual as the fitness function value of the individual.

$$\begin{array}{c} fit\_value=\max\left(z\right)-\sum_{i=1}^{v}\sum_{j=1}^{n}|T_{j}^{a_{ij}}-X_{ij}|\cdot c, \end{array}$$ (15)

where max(*z*) is the maximum of group objective function, $T_{j}^{a_{ij}}$ is the position of the moving channel corresponding to *a*_*ij*_ in matrix ***A***. When *b*_*ij*_ ≠ 0, $T_{j+1}^{a_{ij}}=b_{ij}$, and the other channel positions are unchanged; when *b*_*ij*_ = 0, $|T_{j}^{a_{ij}}-b_{ij}|=0$, $T_{j+1}^{a_{ij}}=T_{j}^{a_{ij}}$, and all the channel positions are unchanged.

#### Individual evaluation: Fitness calculation

According to the abovementioned fitness function, the fitness of the different individuals can be calculated. A smaller fitness value corresponds to a better individual. The order picking information contained in matrix ***B*** is known from the greedy algorithm calculation result. Matrix ***A*** corresponds to only the channel number, and its corresponding channel position may change with the progression of the picking process. Therefore, the calculation process of the fitness function is dynamic, and the individual’s fitness is calculated step by step, with the initial position of the given mobile channel $\left\{T_{1}^{1},T_{1}^{2},\ldots ,T_{1}^{E}\right\}$ and the information of matrices ***A*** and ***B***.

For example, there exist two moving channels, that is, *E* = 2, and the channels are numbered 1 and 2. The initial position of the moving channel is $\left\{T_{1}^{1}=1,T_{1}^{2}=3\right\}$, and the matrices ***A*** and ***B*** are as follows:

$$\begin{array}{c} A=[\begin{array}{cc} \begin{array}{ccc} 2 & 1 & 1\\ 2 & 2 & 2\\ 2 & 2 & 2 \end{array} & \begin{array}{ccc} 2 & 2 & 2\\ 2 & 1 & 1\\ 2 & 1 & 2 \end{array}\\ \begin{array}{ccc} 2 & 1 & 1\\ 2 & 1 & 2\\ 1 & 2 & 2 \end{array} & \begin{array}{ccc} 1 & 2 & 1\\ 1 & 2 & 1\\ 1 & 2 & 1 \end{array} \end{array}],B=[\begin{array}{cc} \begin{array}{ccc} 5 & 1 & 0\\ 3 & 2 & 0\\ 6 & 0 & 3 \end{array} & \begin{array}{ccc} 2 & 0 & 6\\ 4 & 5 & 0\\ 0 & 0 & 6 \end{array}\\ \begin{array}{ccc} 1 & 5 & 0\\ 2 & 0 & 0\\ 3 & 4 & 0 \end{array} & \begin{array}{ccc} 4 & 0 & 6\\ 0 & 6 & 5\\ 1 & 0 & 6 \end{array} \end{array}], \end{array}$$ (16)

The picking process is as follows:

**Step 1:** $\left\{T_{1}^{1}=1,T_{1}^{2}=3\right\}$ and *a*_11_ = 2, *b*_11_ = 5. Since *b*_11_ ≠ 0, the current picking task is to move channel 2 in position 3 to pick up the goods in channel 5, and the time taken is $|T_{1}^{2}-b_{11}|\cdot c=|3-5|\cdot c=2\cdot c$. After the picking task is completed, let $T_{2}^{2}=b_{11}=5$; the positions of the other channels are unchanged, and a new set of mobile channel locations is obtained as $\left\{T_{2}^{1}=1,T_{2}^{2}=5\right\}$;
**Step 2:** $\left\{T_{2}^{1}=1,T_{2}^{2}=5\right\}$ and *a*_12_ = 1, *b*_12_ = 1. Since *b*_12_ ≠ 0, the current picking task is to move channel 1 in position 1 to pick up the goods in channel 1, and the time taken is $|T_{2}^{1}-b_{12}|\cdot c=|1-1|\cdot c=0$. After the picking task is completed, let $T_{3}^{1}=b_{12}=1$, the positions of the other channels are unchanged, and a new set of mobile channel locations $\left\{T_{3}^{1}=1,T_{3}^{2}=5\right\}$ is obtained;
**Step 3:** $\left\{T_{3}^{1}=1,T_{3}^{2}=5\right\}$ and *a*_13_ = 1, *b*_13_ = 0. Since *b*_13_ = 0, the current picking task does not exist; therefore, the time taken is $|T_{3}^{1}-b_{13}|\cdot c=0$. Let $T_{4}^{1}=T_{3}^{1}=1$; the positions of the other channels are unchanged, and a new set of mobile channel positions is obtained as $\left\{T_{4}^{1}=1,T_{4}^{2}=5\right\}$;

Similar steps can be performed until the picking time of each order is known. Subsequently, the total time for the picking up of *v* orders is calculated, and the individual’s adaptive function can be determined. From these results, we can obtain the adaptive functions values of the individuals in the group and arrange them in the increasing order. Without a loss of generality, the results can be presented as in Table 2.

**Table 2:** Individual adaptability.

Individual serial number	1	2	…	*g*
Adaptability	*z*_1_	*z*_2_	…	*z*_*g*_

#### Copying, crossing, and mutation

*Selection operator settings*. In this study, roulette was used to replicate individuals, as shown in Table 3. In view of the fact that the individual fitness difference is small, if only the basic Roulette is chosen, the efficiency of iterative solution process is low. According to the value of fitness function of each individual, it is magnified in different degrees. The higher the fitness, the greater the degree of amplification. The smaller the fitness, the smaller the proportion of amplification. In order to make a more obvious distinction between individuals, it can improve the selection probability of excellent individuals and accelerate the convergence speed of the solution process.

**Table 3:** Roulette method to copy individuals.

Individual serial number: *φ*(1≤*φ*≤*g*)	1	2	…	*g*
Adaptability: *z*_*φ*_	*z*_1_	*z*_2_	…	*z*_*g*_
Accumulated value of fitness: *H*_*φ*_	*H*_1_	*H*_2_	…	*H*_*g*_
Random number between 0 and *H*_*g*_: *R*	*R*_1_	*R*_2_	…	*R*_*g*_

The individuals are arranged according to the serial number, and the fitness value of each individual is obtained correspondingly, and the serial number sequence of fitness values from small to large is obtained. According to the sequence of the serial number, the individual fitness value is enlarged step by step, that is, a gradually increasing positive value is added on the basis of the original fitness. Then, the cumulative value *H*_*φ*_ of the obtained fitness can be calculated as *H*_*φ*_ = *z*_1_ + *z*_2_ + … + *z*_*φ*_. Furthermore, the random number *R* is generated to be between 0 and *H*_*g*_, and the number of *R* is *ξ*, which is a sufficiently large positive integer.

The individual copy rules are as follows:

1. By comparing *R*_1_ to *H*_1_ and *H*_*g*_ one by one, the first value of *H*_*φ*_ that is greater than *R*_1_ is selected, and the individual corresponding to the serial number is copied once;
2. By comparing *R*_2_ to *H*_1_ and *H*_*g*_ one by one, the first value of *H*_*φ*_ that is greater than *R*_2_ is selected, and the individual corresponding to the serial number is copied once;
3. Similar steps are performed until the number of copied individuals reaches *g*; subsequently, the copying process is stopped.

**Fig 5:**
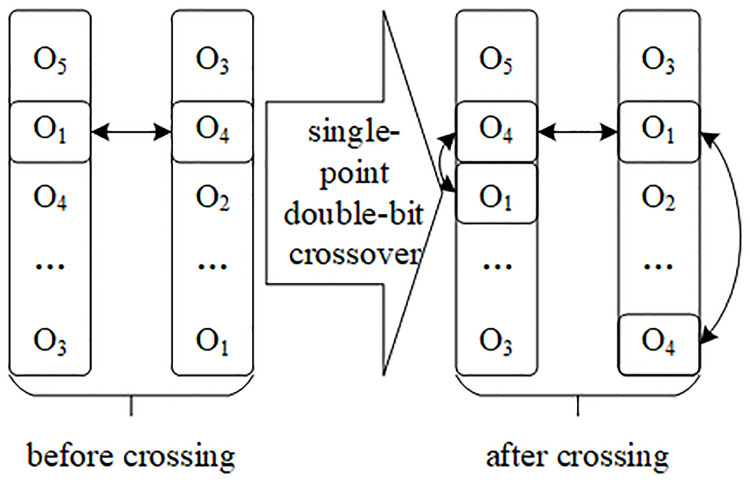
Single-point double-bit crossover operation.

*Crossover operator settings*. On the basis of pairing individual coding information, according to the characteristics of real coding, the mode of “single-point crossover, double-bit exchange” is adopted in the cross operation, as shown in Fig 5. On the basis of traversing the pairing successful order picking sequence set, according to the real number coding information, according to the crossover probability *p*_*c*_, the number of the position corresponding to two real number sequences is exchanged, which is single-point crossover. In addition, it is necessary to transform the number of another position in each sequence which is the same as the number of the crossover position, so that the real number sequence in each order sequence set is still complete and has no repetition after the crossover operation, which is called double-bit exchange. At the same time, the information matrix corresponding to the open channel and the information matrix of the picking channel are transformed in the same way.

*Mutation operator setting* Mutation operation mainly aims at information matrix of open channel and information matrix of picking channel. In the process of traversing each individual’s information, according to mutation probability *p*_*m*_, two rows with the same position of matrix {***A***, ***B***} are selected, and the row is randomized in order, while other individual’s information remains unchanged. In other words, the mutation operation aims at the channel picking sequence of an order in the individual information, and does not change the sequence of the order.

#### Termination conditions

To define the termination condition of the model, the design needs to consider the number of iterations and degree of optimisation. For the maximum number of iterations, the initial setting is 1000 times. For the optimisation degree of the solution, the metric of the optimisation degree needs to be further defined to clarify the termination condition. The algorithm terminates when the maximum number of iterations is reached, or when the required degree of optimisation is reached.

In terms of the measure of the optimisation degree, the minimum expected total time of the virtual order cluster picking can be used as the termination condition, according to the principle that the channel ineffective moving time is the minimum in the greedy algorithm. Considering the number of virtual order clusters and the number of channel locations in the dense mobile rack storage system, combined with the greedy rules, it is reasonable to consider the minimum expected total time as a measure of the degree optimisation.

Let the probability that each channel position is picked be *p*. Since the model is selected for the virtual order cluster based on the order batching,

$$\begin{array}{c} p=\frac{\sum_{i=1}^{v}\sum_{j=1}^{n}b_{ij}}{v\cdot n}, \end{array}$$ (17)

When the total number of channels is *n* and the number of virtual order clusters is *v*, whether each channel position in the *i* − *th* virtual order cluster is selected or not can be regarded as multiple Bernoulli tests. According to the nature of the geometric distribution, the expected values of the lower and upper limits of picking the active interval can be calculated separately.

The expected value of the lower limit of picking the active interval is $b_{i,\cdot }^{min}Y_{\cdot }=\sum_{i=1}^{n}i\cdot p\cdot {\left(1-p\right)}^{i-1}$, and that of the upper limit is $b_{i,\cdot }^{max}Y_{\cdot }=\sum_{i=0}^{n-1}\left(n-i\right)\cdot p\cdot {\left(1-p\right)}^{i}$. The length expectation of the picking active interval for a virtual order cluster is $b_{i,\cdot }^{max}Y_{\cdot }-b_{i,\cdot }^{min}Y_{\cdot }$.

In the case of a single channel, the channel invalid movement time is 0 at least, which is the expected condition of the optimal solution. Therefore, the expected value *E*(*z*) of the total time of picking *v* virtual order clusters is

$$\begin{array}{c} E\left(z\right)=\left(b_{i,\cdot }^{max}Y_{\cdot }-b_{i,\cdot }^{min}Y_{\cdot }\right)\cdot c\cdot v, \end{array}$$ (18)

In the case of multiple channels, the corresponding value can be calculated by converting the channels into multiple single channels. Considering the number of channels to be *E*, the area of the *n* channel positions can be divided into *E* single-channel sub-areas, which are separately calculated and later summed. The number of rack positions in each sub-area is [*n*/*E*], where *E* ≥ 1, and [ ] indicates rounding. The lower and upper limits of the sub-area picking active interval are respectively $b_{i,\cdot }^{min}{Y_{\cdot }}_{sub}=\sum_{i=1}^{\left[n/E\right]}i\cdot p\cdot {\left(1-p\right)}^{i-1}$ and $b_{i,\cdot }^{max}{Y_{\cdot }}_{sub}=\sum_{i=0}^{[n/E]-1}\left(\left[n/E\right]-i\right)\cdot p\cdot {\left(1-p\right)}^{i}$.

Consequently, the optimal time-consuming selection for each sub-area is $(b_{i,\cdot }^{max}{Y_{\cdot }}_{sub}-b_{i,\cdot }^{min}{Y_{\cdot }}_{sub})\cdot c$. In addition, there exist *v* virtual order clusters, and each order cluster has *E* sub-intervals. The optimal situation of the total cost of order picking is expected to be *E*(*z*).

$$\begin{array}{c} E\left(z\right)=\left(b_{i,\cdot }^{max}{Y_{\cdot }}_{sub}-b_{i,\cdot }^{min}{Y_{\cdot }}_{sub}\right)\cdot c\cdot v\cdot E, \end{array}$$ (19)

Subsequently, *E*(*z*) is taken as the standard to be achieved by the degree of optimisation. When the maximum number of iterations reaches 1000 or the objective function value of the solution is *E*(*z*), the iteration is stopped.

**Fig 6:**
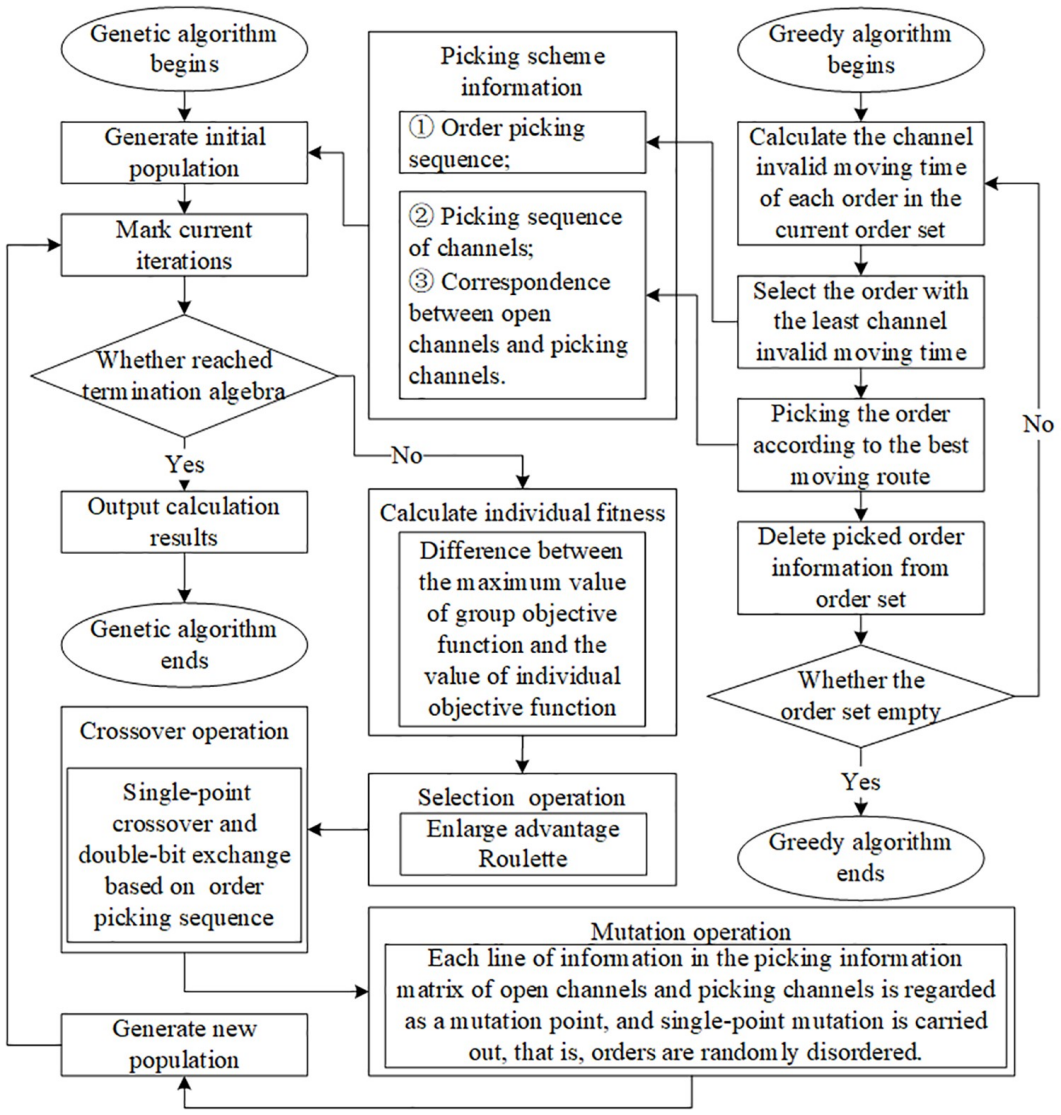
Framework of hybrid genetic algorithm.

According to the above algorithm design, a hybrid genetic algorithm is designed by combining greedy algorithm with genetic algorithm, as shown in Fig 6.

## Example simulation and analysis

### Parameter setting

The text uses the random generation 0 − 1 matrix to obtain the information matrix ***X*** of the order picking as the example data. To compare the different data sizes and different cases, let *n* = 30, *m* = 20 and *n* = 70, *m* = 50, as calculated separately for smaller scale studies (20 orders for 30 channel locations) and larger scale studies (50 orders for 70 channel locations), respectively. In addition, in the case of a large number of orders, the orders are picked by appropriate batching and merging. The probability of being picked before batched of each channel is set as 0.08, and other settings are shown in Table 4.

**Table 4:** Parameter values of examples.

Basic parameters	Number of open channels	Initial channel position	Number of batches	Number of racks	Original order quantity
**Values**	3, 9	‘one’,‘both’,‘middle’,‘uniform’	5,10	30,70	20,50

In terms of the number of shelves and the number of original orders, only two cases are considered: the smaller case (30 channels, 20 orders) and the larger case (70 channels, 50 orders). The values of other parameters are combined with each other, and a total of 32 examples are generated.

The operation parameters of genetic algorithm include: population size (*M*), evolution algebra (*T*), crossover probability (*p*_*c*_), mutation probability (*p*_*m*_). They play an important role in the efficiency of solution and the degree of result optimization, but there is no basis for reasonable selection. Generally, it is necessary to obtain the optimal parameter settings for specific problems through the calculation experiments of sensitivity analysis.

As shown in Table 5, on the basis of setting the general value range [18], the sensitivity analysis of the parameters is carried out with a certain change step. According to the multiple experimental results of different examples, the medium and high frequency parameter values of the optimized parameter set (the parameters corresponding to the first three optimal results) are selected as the final results of parameter values.

**Table 5:** Parameter setting of genetic algorithm.

	Value range	Change step size	Optimal solution parameter set	Final parameter selection
*M*	20–100	40	[100, 100, 100]	100
*T*	100–500	200	[300, 100, 300]	300
*p*_*c*_	0.4–0.99	0.3	[0.99,0.99,0.69]	0.99
*p*_*m*_	0.0001–0.1	0.033	[0.0331,0.0331,0.0331]	0.0331

The above algorithm parameters are combined with each other to generate 108 algorithm parameter combinations.

**Fig 7:**
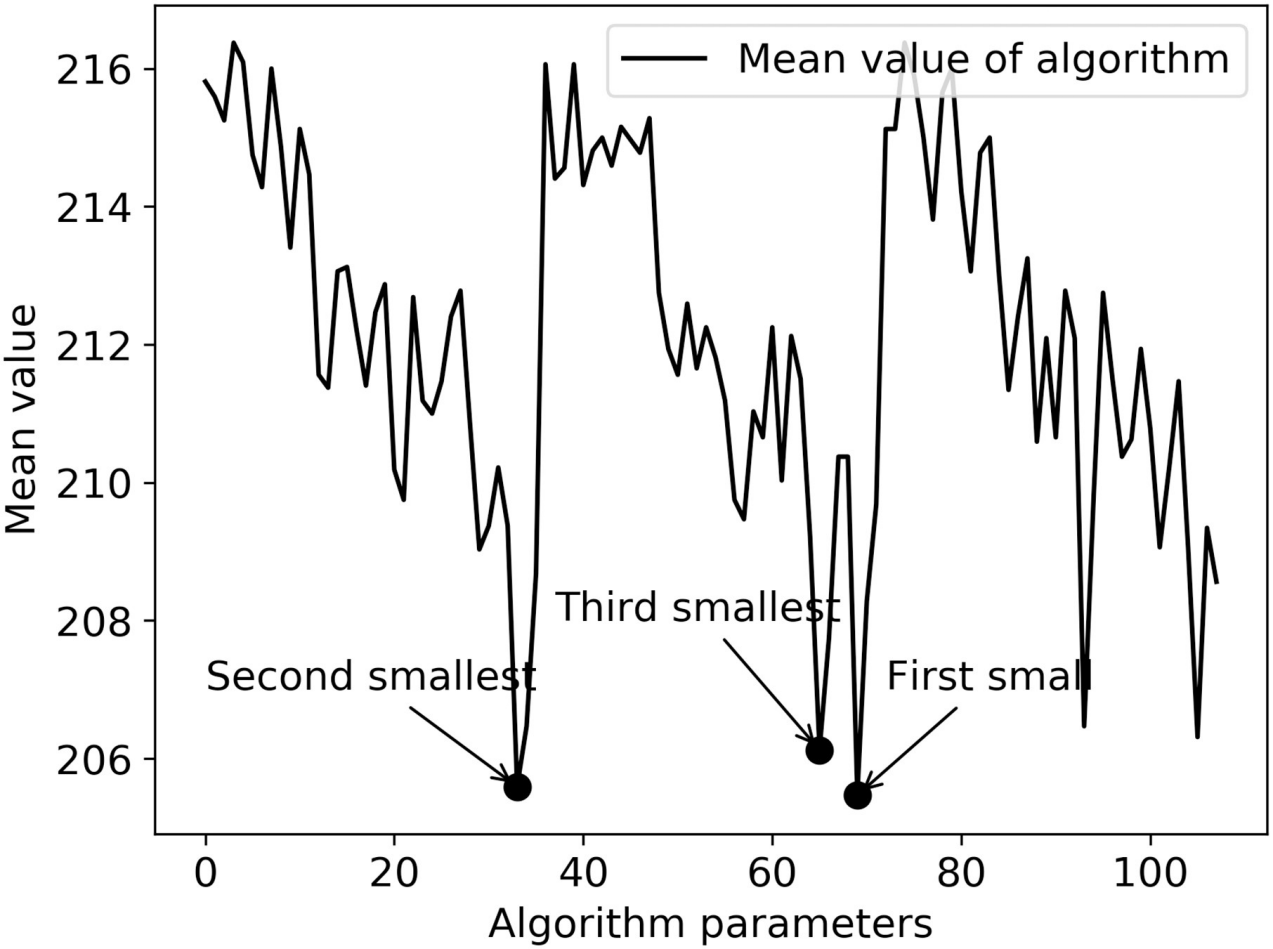
The mean value of the objective function under different parameters.

**Fig 8:**
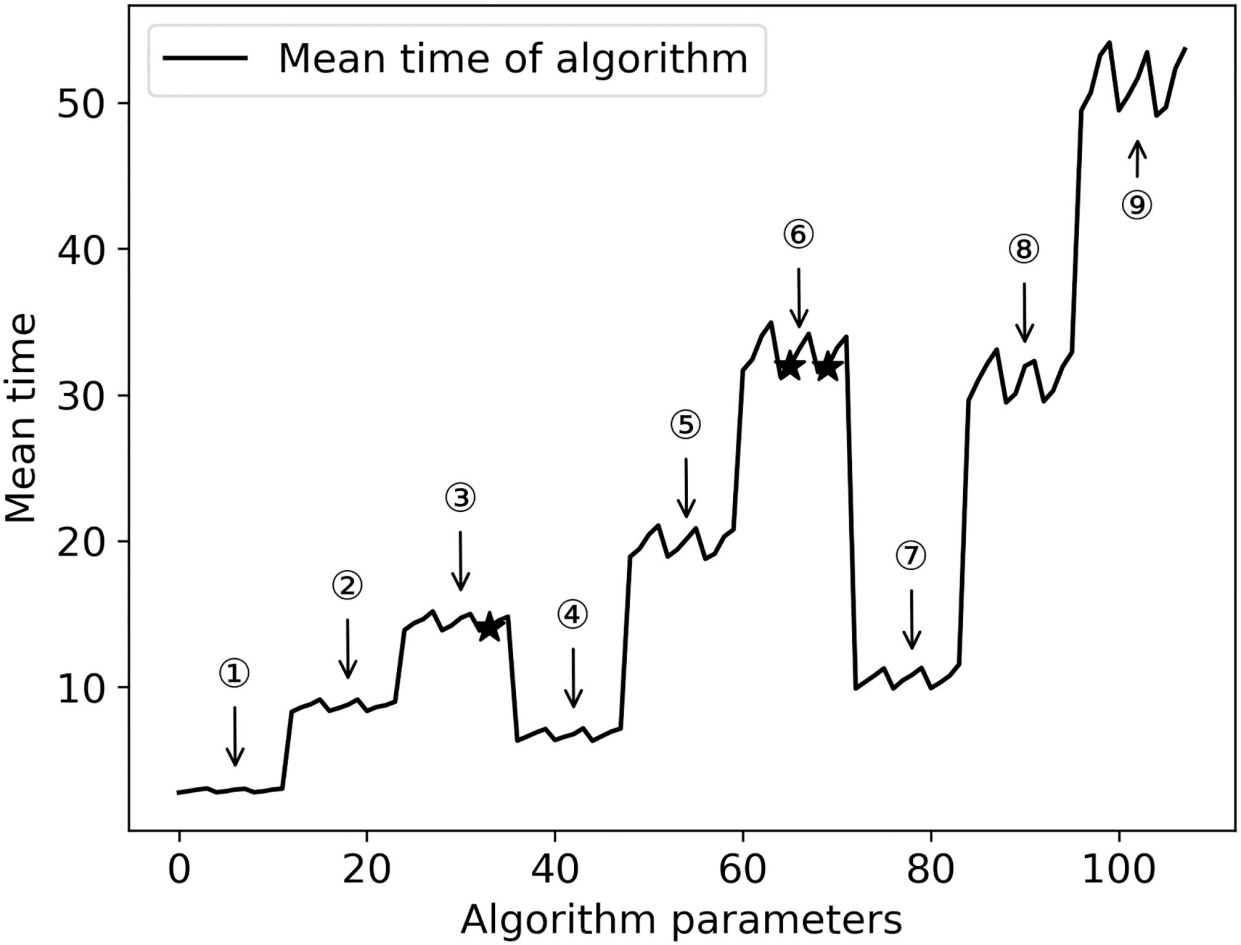
Algorithm execution time cost under different parameters.

The data of 32 examples are calculated under 108 parameter settings, and the value of objective function and the time cost of algorithm operation are analyzed, as shown in Figs 7 and 8. The horizontal axis in Fig 7 shows the combination of various parameter values, and the vertical axis shows the average of the minimum values of the objective function of all examples under different parameter values. The three optimal value points of the objective function have been marked as “•”. The horizontal axis in Fig 8 is the same as that in Fig 7, and its vertical axis represents the average of the minimum running time of all examples, the parameter combination of the three optimal values of the objective function and the algorithm time cost, and the corresponding position in Fig 8 is marked with “★”.

Both Figs 7 and 8 show obvious periodic characteristics. There are three large periodic changes in Fig 7. Parts ① to ⑨ of Fig 8 can also be divided into three large stages according to every three groups, which are: ①-③, ④-⑥ and ⑦-⑨. Through the analysis of algorithm parameters’ setting and cross combination, it can be seen that the three major periodic changes are closely related to the number of iterations and the size of the group. In Fig 7, for the three iterations, when the population size changes from small to large, and with the change of crossover probability and mutation probability, the optimization of the objective function presents a continuous downward trend, while the time cost of the solution process is gradually increasing in the three major stages and the three small parts within each major stage.

Comparing with Figs 7 and 8, in terms of balancing the time cost of solving and the stability of the optimization process, although the parameter combination of the second optimization can reduce the time cost by nearly half, the stability of the parameter combination is poor. Therefore, the parameter values with high optimization degree and high frequency can make the optimization process more stable.

### Example results

According to the analysis results of the determination of algorithm parameters, the optimized parameters are set, and the calculation results of different examples are obtained, as shown in Tables 6 and 7. Among them, the basic parameters (BP) include [number of open channels, distribution of open channels, batch number of orders, rows of racks, quantity of orders]. The values of “distribution of open channels” are “one”, “both”, “middle” and “uniform”, indicating that the initial positions of multiple open channels are at one end, two ends, middle and uniform distribution of mobile racks. Open access location (OAL) is the number of the initial location of open channels. Channel picking probability (CPP) is the average probability that each channel in the virtual order cluster is picked after batched. The theoretical expected optimal value (TEOV) represents the minimum order picking cost (i.e. the minimum objective function value) that can be achieved in theory, which can be used to measure the effectiveness of the algorithm. However, there are differences between the theoretical expected optimal value and the actual calculation results, so the theoretical expected optimal value can not always be achieved. It should be noted that the calculation of the theoretical expected optimal value only considers the picking probability of the channel, rows of racks and the number of open mobile channels, and does not consider the initial position of mobile channels. Therefore, the optimal value of theoretical expectation is not an absolute measure. Because the problem in this study is NP hard and it is difficult to measure the degree of optimization of the solution, the value is taken as a reference in a relative sense, and the actual calculation result may be higher or lower than the theoretical expected optimal value. In order to further measure the effectiveness of the algorithm, the results of various algorithms will be compared to further verify the optimization degree of the solution and the effectiveness of the algorithm. Distance greedy algorithm (DGA), general genetic algorithm (GGA) and hybrid genetic algorithm (HGA) successively represent the calculation results obtained by greedy algorithm, genetic algorithm based on common iterative rules and hybrid genetic algorithm.

**Table 6:** Results of small scale examples.

	BP	OAL	CPP	TEOV	GGA	DGA	HGA
1	[3, ‘one’, 5, 30, 20]	2, 4, 6	33.33%	80	215	85	85
2	[3, ‘one’, 10, 30, 20]	2, 4, 6	17.33%	84	220	95	**55**
3	[3, ‘both’, 5, 30, 20]	2, 4, 28	26.67%	66	122	**77**	77
4	[3, ‘both’, 10, 30, 20]	2, 4, 28	16.67%	80	194	**72**	**72**
5	[3, ‘middle’, 5, 30, 20]	11, 13, 17	31.33%	76	188	80	**50**
6	[3, ‘middle’, 10, 30, 20]	11, 13, 17	15.00%	71	214	78	78
7	[3, ‘uniform’, 5, 30, 20]	9, 22, 28	36.00%	85	197	**65**	**65**
8	[3, ‘uniform’, 10, 30, 20]	23, 26, 27	15.33%	73	201	86	86
9	[9, ‘one’, 5, 30, 20]	2, 4, 6, 8, 10, 12, 14, 16, 18	39.33%	35	304	54	54
10	[9, ‘one’, 10, 30, 20]	2, 4, 6, 8, 10, 12, 14, 16, 18	13.67%	25	168	44	**23**
11	[9, ‘both’, 5, 30, 20]	2, 4, 6, 8, 10, 22, 24, 26, 28	34.67%	31	194	36	**29**
12	[9, ‘both’, 10, 30, 20]	2, 4, 6, 8, 10, 22, 24, 26, 28	17.00%	31	223	35	**5**
13	[9, ‘middle’, 5, 30, 20]	5, 7, 9, 11, 13, 17, 19, 21, 23	26.67%	24	143	39	39
14	[9, ‘middle’, 10, 30, 20]	5, 7, 9, 11, 13, 17, 19, 21, 23	16.67%	30	201	41	**15**
15	[9, ‘uniform’, 5, 30, 20]	3, 5, 13, 13, 16, 16, 18, 22, 27	34.00%	31	202	35	**27**
16	[9, ‘uniform’, 10, 30, 20]	3, 6, 11, 14, 18, 19, 19, 21, 24	12.00%	22	155	29	**19**

**Table 7:** Results of large scale examples.

	BP	OAL	CPP	TEOV	GGA	DGA	HGA
1	[3, ‘one’, 5, 70, 50]	2, 4, 6	79.14%	322	2795	304	**298**
2	[3, ‘one’, 10, 70, 50]	2, 4, 6	41.00%	574	3428	547	**532**
3	[3, ‘both’, 5, 70, 50]	2, 4, 68	82.29%	324	3174	308	**308**
4	[3, ‘both’, 10, 70, 50]	2, 4, 68	39.43%	568	2984	491	**487**
5	[3, ‘middle’, 5, 70, 50]	31, 33, 37	90.29%	327	2815	**307**	**307**
6	[3, ‘middle’, 10, 70, 50]	31, 33, 37	40.00%	570	3362	**556**	**556**
7	[3, ‘uniform’, 5, 70, 50]	42, 56, 66	77.43%	321	3093	321	321
8	[3, ‘uniform’, 10, 70, 50]	31, 46, 57	41.86%	577	3531	562	**513**
9	[9, ‘one’, 5, 70, 50]	2, 4, 6, 8, 10, 12, 14, 16, 18	81.43%	249	2565	269	269
10	[9, ‘one’, 10, 70, 50]	2, 4, 6, 8, 10, 12, 14, 16, 18	39.29%	303	3385	424	424
11	[9, ‘both’, 5, 70, 50]	2, 4, 6, 8, 10, 62, 64, 66, 68	83.71%	252	2783	**242**	**242**
12	[9, ‘both’, 10, 70, 50]	2, 4, 6, 8, 10, 62, 64, 66, 68	39.86%	307	3117	353	353
13	[9, ‘middle’, 5, 70, 50]	25, 27, 29, 31, 33, 37, 39, 41, 43	81.43%	249	2736	251	251
14	[9, ‘middle’, 10, 70, 50]	25, 27, 29, 31, 33, 37, 39, 41, 43	36.14%	280	2884	324	324
15	[9, ‘uniform’, 5, 70, 50]	2, 6, 13, 44, 49, 52, 66, 67, 69	78.86%	246	2577	217	**213**
16	[9, ‘uniform’, 10, 70, 50]	2, 4, 10, 13, 26, 33, 37, 51, 63	43.71%	333	3758	354	354

It can be seen from the above calculation results that in 62.5% of small-scale cases and 56.25% of large-scale cases, the results of HGA are better than DGA and GGA, and the optimization degree exceeds TEOV. The results of DGA are the same as that of HGA in only 18.75% of the cases, but GGA is not good in all the cases. It can be seen that the HGA obtained by combining DGA with GGA can better inherit the excellent genes of the parent solution, and further improve the optimization degree of the results by using the selection operator of amplifying dominant roulette, the crossover operator of “single-point crossover, double-bit exchange” and the mutation operator of “single-point mutation, random shuffle”.

The order batch quantity is an important factor affecting the picking efficiency. As shown in Table 8, when the scale of examples is small, a smaller allocation quantity can not improve the picking efficiency, but a larger allocation quantity can make the picking result better. However, when the sample size is large, the effect of order batch quantity on the picking efficiency becomes very obvious. Less order batch quantity can greatly improve the picking efficiency, while when the order batch quantity is large, the order picking efficiency decreases significantly.

**Table 8:** Analysis of order picking efficiency in different cases based on HGA algorithm.

Calculation results of HGA		Small scale examples		Large scale examples	
		3 open channels	9 open channels	3 open channels	9 open channels
5 order batches	one	85	54	298	269
	both	77	29	308	242
	middle	**50**	39	**307**	251
	uniform	65	**27**	321	**213**
10 order batches	one	**55**	23	532	424
	both	72	**5**	**487**	353
	middle	78	15	556	**324**
	uniform	86	19	513	354

In addition, further analysis of the data in Table 8 shows that the initial distribution position of open channels also has an impact on the order picking efficiency. When the order picking positions are evenly distributed in mobile racks, in 62.5% of the cases, the worst results are obtained when open channels’ positions are located at one end of mobile racks; in 87.5% of the cases, the better results are obtained when other distribution methods are used except that open channels are concentrated at one end. When open mobile channels are distributed in the middle of racks, 37.5% of the results are better than other distributions; when open mobile channels are distributed at both ends or evenly, 25% of the results are better than other distributions.

### Suggestion and analysis

#### Algorithm selection in different situations

As shown in Table 9, the calculation results of HGA are compared with those of GGA and DGA respectively. The calculation example scale refers to the number of racks and the number of orders. “HGA vs. GGA” means to divide the difference between the calculation results of GGA and HGA by the calculation results of GAA, and so on, other relative optimization indexes can be calculated. Compared with GGA and DGA, the optimization degree of HGA is 86.37% and 34.21% when the scale of the example is small and the number of channels is large, so the hybrid genetic algorithm should be preferred; when the scale of the example is small and the number of open channels is small, the relative optimization degree of HGA decreases. When the scale of examples is large, the optimization degree of HGA is more than 86% relative to GGA, but less than 2% relative to DGA. Considering that the calculation time cost of HGA is much higher than DGA, DGA is suitable for calculation. In addition, the optimization degree of GGA is far lower than other algorithms in all cases, so the algorithm is not suitable for any case.

**Table 9:** Comparison of relative optimization degree of different examples and algorithms.

	Small scale examples				Large scale examples			
	3 open channels		9 open channels		3 open channels		9 open channels	
	HGA vs. GGA	HGA vs. DGA	HGA vs. GGA	HGA vs. DGA	HGA vs. GGA	HGA vs. DGA	HGA vs. GGA	HGA vs. DGA
1	60.47%	0.00%	82.24%	0.00%	89.34%	1.97%	89.51%	0.00%
2	75.00%	42.11%	86.31%	47.73%	84.48%	2.74%	87.47%	0.00%
3	36.89%	0.00%	85.05%	19.44%	90.30%	0.00%	91.30%	0.00%
4	62.89%	0.00%	97.76%	85.71%	83.68%	0.81%	88.68%	0.00%
5	73.40%	37.50%	72.73%	0.00%	89.09%	0.00%	90.83%	0.00%
6	63.55%	0.00%	92.54%	63.41%	83.46%	0.00%	88.77%	0.00%
7	67.01%	0.00%	86.63%	22.86%	89.62%	0.00%	91.73%	1.84%
8	57.21%	0.00%	87.74%	34.48%	85.47%	8.72%	90.58%	0.00%
Means	62.05%	9.95%	86.37%	34.21%	86.93%	1.78%	89.86%	0.23%

#### Batching strategy under different storage capacity

According to the analysis of HGA calculation results of different order batches in Table 8, when the storage capacity of mobile racks is small, the number of mobile racks is less or the storage location of mobile racks is less due to its short length, there is no need to do too much batch processing, and the better results can be obtained by directly picking according to the received original order information as far as possible. When the number of mobile racks is large and the probability of being picked in the corresponding channel of each storage rack is high, the received orders should be processed in batches as far as possible, and then the combined virtual order cluster information should be used for picking. However, it should be noted that the implicit premise of this case is that the picking probability of each channel obeys uniform distribution, and there is no distinction between the specific location of goods on racks. If more complex cases are considered, further research is still needed.

#### Influence of initial position of open channels

Through the analysis of the example data in Table 8, we can see that the initial location distribution of the open channels should be concentrated in the middle of the mobile rack as far as possible, and avoid concentrated in one end of the mobile rack. In addition, the number of batches will affect the picking probability of each storage rack. When the order is divided into fewer batches, the initial position of the open channels should be located in the middle of the storage position of mobile racks, or evenly distributed in different positions of the whole row of mobile racks. When the order is divided into many batches, the initial position of mobile racks should be located at both ends of the whole row of mobile racks as far as possible.

## Conclusions and prospects

In this work, the mathematical model for a dense mobile rack storage system is established, and a solving algorithm is designed to optimize the order picking. Based on the simulations of examples with different scales, the nature and characteristics of this problem are further analysed. The main characteristics are as follows:

1. Based on the hierarchical clustering method, the order is processed in batches, and the picking model is established on the basis of order batching, which improves the picking efficiency of the dense mobile rack storage system.
2. The hybrid genetic algorithm is designed by combining the greedy algorithm and the genetic algorithm. Compared with those of the simple genetic algorithm, the efficiency and solution optimisation of hybrid the genetic algorithm are improved.
3. The index of the “objective function optimal expectation value” is proposed as the termination condition and used as a measure of the satisfactory solution when using the hybrid genetic algorithm to ensure that the optimisation degree of the solution can be more clearly evaluated.
4. By performing the simulation of different scales, the influence of various parameters on the nature of the problem is further analysed, and the application of the greedy algorithm, hybrid genetic algorithm and simple genetic algorithm is analysed.

The abovementioned research results indicate that the order picking problem of dense mobile racks is complex, especially when the problem scale is large, and critical challenges arise in the form of the efficiency of the algorithm and optimisation degree of the solution. Therefore, improving the algorithm is an important future research direction in this domain. In addition, as the research progresses, more practical conditions need to be considered to adapt the model to different situations. For example, the construction of a dual objective model [19] considering the minimization of the number of personnel and the total cost of picking, and the design of a multi-objective genetic algorithm [20], one can consider the number of picks per channel and travel time of the picker (or picking vehicle), among other factors. Considering these aspects, the number of variables contained in the model will increase, thereby increasing the model complexity. Furthermore, the algorithm can be improved by combining more factors to solve the problem, such as using the multi-stage hybrid solution method of clustering, dynamic programming and heuristic algorithm to solve the total cost minimization problem [21].

In summary, there exists considerably research space for the order picking problem of dense mobile racks, which still needs to be optimised in many aspects. Furthermore, the conclusions obtained by performing in-depth research on this issue could provide valuable references for other types of compact and dense storage systems.
